# Genotypic Variability on Grain Yield and Grain Nutritional Quality Characteristics of Wheat Grown under Elevated CO_2_ and High Temperature

**DOI:** 10.3390/plants10061043

**Published:** 2021-05-21

**Authors:** Emilio L. Marcos-Barbero, Pilar Pérez, Rafael Martínez-Carrasco, Juan B. Arellano, Rosa Morcuende

**Affiliations:** Institute of Natural Resources and Agrobiology of Salamanca (IRNASA), Consejo Superior de Investigaciones Científicas (CSIC), 37008 Salamanca, Spain; emiliol.marcos@irnasa.csic.es (E.L.M.-B.); pili2013@gmail.com (P.P.); rafael.mcarrasco@gmail.com (R.M.-C.); juan.arellano@irnasa.csic.es (J.B.A.)

**Keywords:** wheat, elevated CO_2_, temperature, grain yield, grain protein concentration, mineral nutrients, phenolic compounds, starch

## Abstract

The progressive rise in atmospheric CO_2_ concentrations and temperature associated with climate change is predicted to have a major impact on the productivity and quality of food crops. Therefore, food security is highly dependent on climate change. Following a survey with 60 bread wheat genotypes, here we investigated the genetic variation in grain yield and nutritional quality among 10 of these genotypes grown under elevated CO_2_ and temperature. With this purpose, the biomass production, grain yield-related traits, the grain concentration of starch, total protein, phenolic compounds, and mineral nutrients, together with the total antioxidant capacity, were determined. Variation among genotypes was found for almost all the studied traits. Higher grain and ear numbers were associated with increased grain yield but decreased grain total protein concentration and minerals such as Cu, Fe, Mg, Na, P, and Zn. Mineral nutrients were mainly associated with wheat biomass, whereas protein concentration was affected by plant biomass and yield-related traits. Associations among different nutrients and promising nutrient concentrations in some wheat genotypes were also found. This study demonstrates that the exploration of genetic diversity is a powerful approach, not only for selecting genotypes with improved quality, but also for dissecting the effect of the environment on grain yield and nutritional composition.

## 1. Introduction

The world population is expected to grow from the current 7.6 billion to 9.7 billion by 2050, while more than 820 million people still suffer from undernourishment, and over 700 million are exposed to severe levels of food insecurity [1]. Therefore, a major challenge for food security is to increase crop yield and quality to meet the growing global demand for food at a time of unprecedented climatic variability [2]. The major driver for the current changes in climate is the rapid increase in atmospheric carbon dioxide (CO_2_) concentration due to anthropogenic activities [3]. Since the Industrial Revolution, the global atmospheric CO_2_ concentration has increased from 280 ppm to currently exceed 410 ppm [4], and it is expected to rise even further to levels of ~1000 ppm by the end of this century causing significant changes in global temperature. Over the past century, the mean global air temperature has risen about 0.8 °C, and it is predicted to increase by an average of 2.6–4.8 °C throughout this century with more frequent occurrences of extreme climatic events such as heatwaves, drought, and heavy rainfall [5]. These environmental changes, which often co-occur, will directly or indirectly affect both productivity of agricultural plants and crop quality [6].

Wheat (*Triticum aestivum* L.), as one of the most important crops worldwide and a major staple food in temperate countries, provides over 20% of the total calories and 22% of the total protein in the human diet [7]. Wheat grains are important sources of carbohydrates, proteins, amino acids, lipids, and minerals, as well as phytochemicals and dietary fiber components [8], which determine the dietary nutritional value and important end-use quality characteristics. In addition, grain protein concentration and composition are crucial characteristics determining the economic value of a wheat crop and the functional quality of the flour. They are also responsible for conferring the viscosity and elasticity properties in the dough for bread production and other products [8,9].

Elevated CO_2_ can positively impact C_3_ food crop production such as wheat by stimulating photosynthetic carbon gain, and consequently increasing crop biomass and yield [10]. The growth stimulation is a result of both enhanced photosynthesis, but also improved water use efficiency due to reduced stomatal conductance. However, as in many other plant species, long-term exposure of wheat plants to elevated CO_2_ leads to a down-regulation of photosynthetic capacity, accompanied by a reduction in Rubisco activity and content, and accumulation of carbohydrates and lower plant protein and N concentrations [10,11,12,13,14,15,16]. A meta-analysis on wheat, rice, maize, sorghum, pea, and soybean grown under field conditions at elevated CO_2_ reported that C_3_ crops had lower grain protein concentration, whereas C_4_ crops were less affected [17]. In line with this, a decrease in total grain protein concentration with elevated CO_2_ was reported in wheat [9,18,19], which may compromise the grain nutritional quality with serious consequences for public health in countries where the main protein source comes from C_3_ grains. Apart from a decrease in grain protein concentration, CO_2_ enrichment may also lead to a reduction not only in macroelements such as Na, Ca, Mg, and S in the wheat grain [9,20] depending on the variety [20], but also in microelements such as Fe and Zn [17,18,19]. Such impoverishment in grain mineral composition might have deleterious effects on human health.

In the Mediterranean areas, where wheat is more commonly cultivated, trends in increasing growing season temperatures have already been reported [21]. Further rise in temperature is likely to put both the production and the quality of grains at increasing risk [22,23]. Exposure to higher temperatures during the wheat reproductive phase is more harmful than during the vegetative phase due to the direct impact of temperature on grain number and weight, with the grain weight being the most sensitive yield component [21]. In these areas, the maturity crop stage coincides with higher temperatures, which accelerate crop development while shortening the duration of the grain filling period and the starch biosynthesis and deposition. As a consequence, altered grain quality is associated with smaller grains [21] and higher protein content. Thus, the nutritional composition and quality of wheat grain are not stable. Indeed, it depends on both genetic variability and the environmental conditions where wheat is grown [23].

The prediction of future increased average temperatures under CO_2_ enrichment represents a significant challenge for delivering grain of consistent quality, particularly in more vulnerable regions such as the Mediterranean. Therefore, improved crop varieties will be required to ensure food security in the face of a growing worldwide population. A promising approach might be exploiting genotypic variability in the ability to maintain grain yield and quality while simultaneously adapting to global environmental change. Grain yield of wheat has increased significantly worldwide from the early 1960s, coinciding with the Green Revolution and the introduction of semi-dwarfing genes. The latter allowed a reduction in plant size, which brought together an increase in the harvest index, the number of grains per unit area and grain yield, as well as improved grain protein content [24]. In spite of these grain improvements, a global trend towards lower grain quality in highly yielding agronomical conditions and modern cultivars has been reported since breeders are mainly selecting for grain yield but not grain quality [25]. To date, most of the studies have focused on the impact of elevated CO_2_ and temperature on wheat productivity or quality under controlled and field conditions [9,20,23,26,27]. However, little attention has been paid to the combined effects of CO_2_ enrichment and high temperature, together with the genotypic diversity, to explore the impact not only on grain yield but also on nutritional quality for human health. Based on the assumption that temperatures will not be uniform over the day and the growing season, we have conducted an experiment in controlled environment chambers to gain full control of climatic parameters for the simulation of a typical natural Mediterranean-like environment from the region of Salamanca (Spain) [11,28,29]. The aim of this work was to investigate the genetic variation in yield performance and grain nutritional quality traits across different bread wheat genotypes grown under elevated CO_2_ and temperature. This study was focused on 10 out of 60 wheat lines compared in a precedent screening under the same environment. With this main objective, data for dry weight biomass production, grain yield, and yield components, together with the total antioxidant capacity (TAC) and the concentration of starch, total protein (TP), total phenolic compounds (TPhC), and mineral nutrients in the grain were determined. The genotypic variability for plant biomass, grain yield, and grain nutritional traits was evaluated, and their correlations were also explored. Our study provides valuable information for the improvement of grain yield and grain quality under a complex climate change scenario.

## 2. Results

### 2.1. Wheat Production and Grain Yield

In the present study, the highest aboveground biomass and grain yield were found for genotypes 41, 43, and 61, followed by genotype 95, with mean values for the productivity of 10.27, 10.83, 9.98, and 9.48 g per plant (Table 1), respectively. Furthermore, genotypes 41, 43, and 61 also exhibited the greatest grain number, ear number, harvest index (HI), chaff biomass and, together with genotype 94, the highest grain number per ear (GNE). In addition to genotype 150, genotype 8 had the lowest aboveground biomass, grain yield and HI, as well as the lowest stalk weight along with genotype 23 and the smallest grain number per ear and per plant together with genotypes 74 and 76. The lowest ear number was found for genotypes 23, 94, 95, and 150. Genotype 95, in addition to genotypes 23, 74, and 76, also had the greatest grain weight, and, together with genotype 43, the highest grain yield per ear (GYE). Genotypes 41, 61, 94, and 150 exhibited the lowest grain weight, whereas genotypes 8, 41, and 76 had the shortest GYE.

### 2.2. Wheat Grain Nutritional Quality

Variation for most of the nutritional quality traits analyzed was found across the studied genotypes. Thus, the grain starch concentration did not change among genotypes (Table 2). The TP concentration was higher in the grain of genotypes 8, 23, and 150, and lower for genotypes 43 and 95. In turn, the maximum TAC and TPhC concentration in the grain were found for genotypes 23, 41, 43, and 95, with the minimum TAC for genotypes 8 and 150 and the TPhC concentration for genotypes 74 and 150. A similar pattern of changes was found for the B, Cu, Fe, Mg, and Zn concentrations (Table 3), although some of these changes were not statistically significant. Genotypes 8 and 23 exhibited the greatest values (as well as genotype 150 for the B and Zn concentrations) and genotypes 43 and 76 (together with genotypes 61 and 150 for Fe and Mg) the lowest. In contrast, genotype 41 exhibited the highest concentrations of Ca, K, P, and S, alone or together with genotypes 95 (for Ca and S) or 61 (K), while the lowest concentrations were found in the grain of genotype 150, along with genotype 8 for the Ca concentration and genotypes 8 and 23 for the K concentration.

### 2.3. Genotypic Characterization

**Figure 1:**
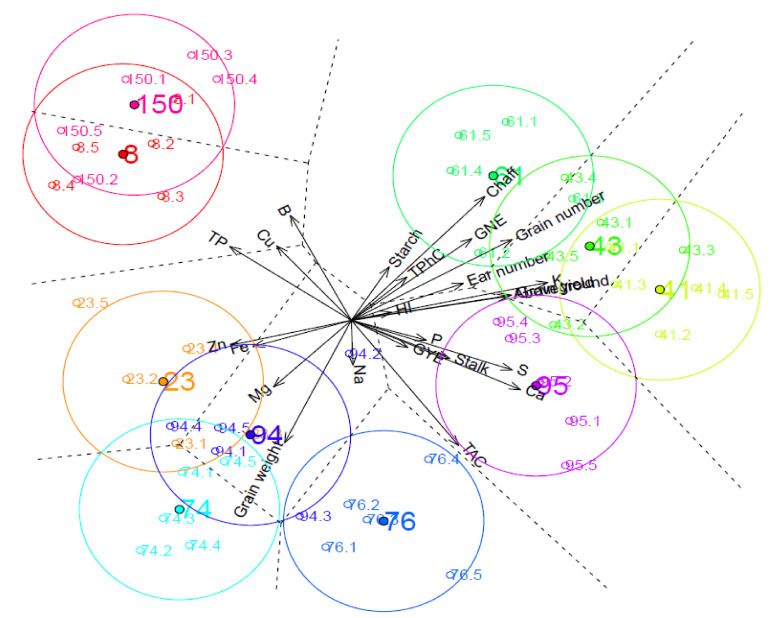
Canonical biplot for the wheat production, grain yield, non-mineral and mineral quality components of 10 wheat genotypes grown under elevated CO_2_ and high temperature. *GNE*: grain number ear^−1^; *GYE*: grain yield ear^−1^; *HI*: harvest index; *TAC*: total antioxidant capacity; *TP*: total protein; *TPhC*: total phenolic compounds. Individuals are represented by empty dots, labelled based on their genotype and numbered as one of the five replicates (*n* = 5) for each group. Filled points represent projections of the averages of the genotypes on the biplot with confidence circles based on Bonferroni’s post-hoc tests. Dotted lines indicate the boundaries of the regions for each group on the Voronoi diagram.

The canonical biplot (CB; Figure 1) shows the maximum differences among genotypes and the traits responsible for this discrimination, while the correlations between the traits studied and the canonical axes are described in Table 4. The first two dimensions of the CB collected a cumulative variance of 53.18% (Table S1), with the first dimension positively associated with wheat production (aboveground, chaff, and stalk biomasses) and most of the grain yield components (grain yield, grain and ear numbers, GNE, GYE, and HI), as well as with K, Ca, and S concentrations, but negatively correlated with TP, grain weight, and grain Zn, Fe, Mg, Cu, and B concentrations. By contrast, the grain number per ear and per plant, chaff weight and TP, B, and Cu concentrations were the major traits positively correlated with the second dimension of the CB, while TAC, grain weight, and Ca, Mg, S, and Na concentrations were negatively associated. As a result, genotypes 41, 43, 61, and 95 were associated with improved grain yield, aboveground biomass and K concentration. However, in comparison with genotype 95, genotypes 41, 43, and 61 were also associated with higher chaff dry weights, ear number and grain number per ear and per plant. In concordance with data reported from ANOVA (Table 3), both genotypes 41 and 95 were also associated with improved S and Ca concentrations, whereas genotypes 61 and 95 had the greatest GYE and TAC. Furthermore, genotypes 8, 23, 74, and 150 were related to improved Fe and Zn concentrations in the grain. While genotypes 8 and 150 were characterized by improved grain TP, B, and Cu concentrations and low TAC, genotypes 23 and 74 were associated with higher grain weight and Mg concentration.

### 2.4. Wheat Production, Grain Yield, and Nutritional Quality Traits

**Figure 2:**
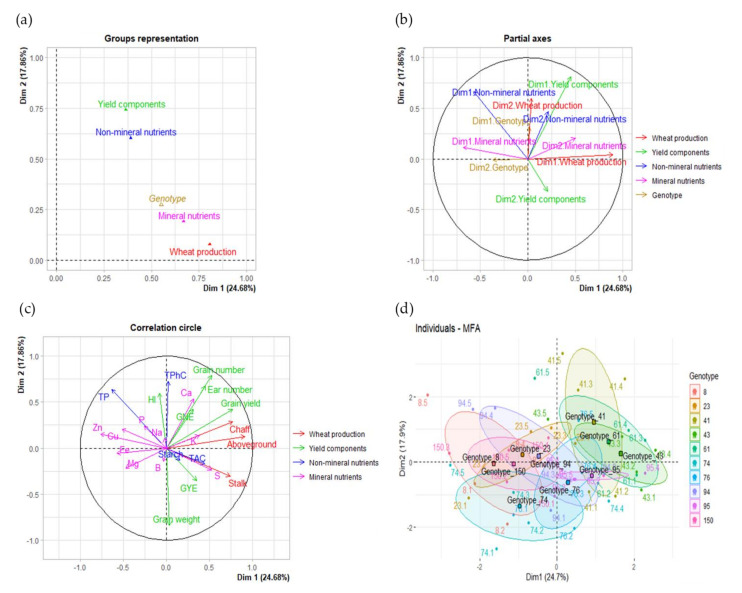
Multiple factorial analysis for the wheat production, grain yield, non-mineral and mineral quality components of 10 wheat genotypes grown under elevated CO_2_ and high temperature. *Dim.*: Dimension; *GNE*: grain number ear^−1^; *GYE*: grain yield ear^−1^; *HI*: harvest index; *TAC*: total antioxidant capacity; *TP*: total protein; *TPhC*: total phenolic compounds. *Genotype* is the group based on a categorical variable specifying the genotypic identity of each sample. The vegetative biomass, grain yield and nutritional quality traits were split up into four groups: *Wheat production* (aboveground, stalk, and chaff biomasses), *Yield components* (grain yield, grain number, ear number, grain weight, grain yield ear^−1^, grain number ear^−1^, and harvest index), *Non-mineral nutrients* (starch, total protein, total phenolic compound concentrations, and total antioxidant capacity) and *Mineral nutrients* (B, Ca, Cu, Fe, K, Mg, Na, P, S, and Zn mineral concentrations). (**a**) The *group representation plot* illustrates the correlation between the variable groups and the supplementary group with axes; (**b**) The *partial axes plot* shows the relationship between the main axes of the MFA and the first two dimensions of each group. (**c**) The *correlation circle plot* represents the correlation of traits with the MFA axes. (**d**) The *individual plot* exhibits the position of individuals in the MFA by genotype variation. *Triangles* indicate the relative position of each group with the axes. *Dots* represent individuals. *Squares* represent group mean points for categorical variables. Ellipses around each genotype (**d**) were added.

A multiple factorial analysis (MFA) was performed to study the existing relationships among vegetative biomass, grain yield components, and non-mineral and mineral nutrients in grain. The variables studied were split up into four groups: *Wheat production*, *Yield components*, *Non-mineral nutrients,* and *Mineral nutrients*. A factor group *Genotype* was also employed in order to analyze the impact of genotypic variation. Figure 2a shows the correlations of the factor group *Genotype* and the four variable groups with the first two dimensions of the MFA. In turn, Table 5 shows numerically both, the correlation and the contribution of the groups and the traits to the dimensions.

**Figure 3:**
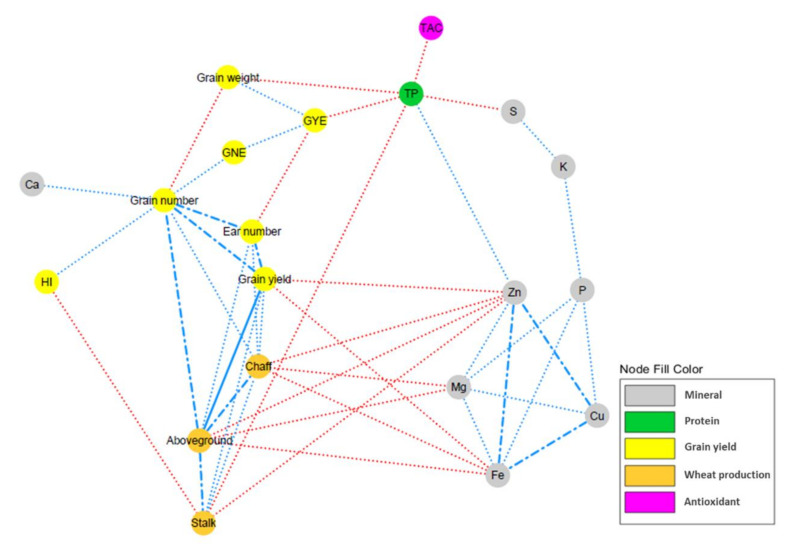
Overview of the correlation network for the wheat production, grain yield and nutritional quality traits of 10 wheat genotypes grown under elevated CO_2_ and high temperature. *GNE*: grain number ear^−1^; *GYE*: grain yield ear^−1^; *HI*: harvest index; *TAC*: total antioxidant capacity; *TP*: total protein; *TPhC*: total phenolic compounds. The different traits (nodes) were classified by colors according to their grain yield or nutritional quality nature (see legend). Edges stand for a Spearman’s correlation *r* ≥ |0.45|, split up as dot (|0.65| > *r* ≥ |0.45|), dash and dot (|0.85| > *r* ≥ |0.65|) or solid (|1| > *r* ≥ |0.85|) line types. Blue edges indicate positive correlation whereas red edges implicate negative correlation.

The first two dimensions of the MFA collected a cumulative variance of 42.54%, with the variable group *Wheat production* as the main contributing group and the more correlated with the first dimension of the MFA (36.17, 0.81; Table 5), followed by the *Mineral nutrients* (29.99, 0.67), *Non-mineral nutrients* (16.35, 0.37) and *Yield components* (16.35, 0.37) groups. In contrast, the *Yield components* group contributed better to the second dimension (46.01, 0.74; Table 5) than the *Non-mineral nutrients* (37.41, 0.60), *Mineral nutrients* (11.80, 0.19), and *Wheat production* (4.79, 0.08). Furthermore, the traits that better contributed to the first dimension were the aboveground biomass, the TP, and the chaff and stalk weights (15.26, 11.25, 10.83, and 10.07; Table 5), while TPhC, TP, grain weight and grain number were the most contributing variables for the second dimension of the MFA (20.05, 15.55, 12.37, and 10.91, respectively). The plots for the partial axes (Figure 2b) and the correlation circle (Figure 2c) showed an opposite distribution for the first dimensions of groups *Mineral nutrients* and *Wheat production*, together with the second dimension of the *Mineral nutrients*. Similarly, the first dimension of the *Non-mineral nutrients* and the *Yield components* showed an opposite association with the first axis of the MFA but a similar correlation with the second dimension of the plot. Thus, TP and B, Cu, Fe, Mg, Na, P, and Zn mineral concentrations in the grain were associated with genotypes 8, 23, 74, and 150 in the individuals MFA plot (Figure 2d), whereas the aboveground, chaff, and stalk biomasses, grain yield, grain and ear number, GNE, and Ca, K, and S concentrations in the grain were mostly associated with genotypes 41, 43, 61, and 95. Moreover, the aboveground, stalk and chaff biomasses were the most positively correlated with the first dimension of the MFA (0.91, 0.77, and 0.74, respectively; Table 5) together with grain yield (0.77). The grain number (0.53), ear number (0.45), GYE (0.35), and GNE (0.31) were also positively correlated with the first axis of the plot, together with S (0.53), K (0.38), Ca (0.32), and TAC (0.45). In contrast, the TP, Zn, Fe, Cu, Mg, and P concentrations were negatively correlated with the first dimension of the MFA in a range from −0.76 to −0.26. These negative correlations of plant production and grain yield components to TP and mineral concentrations, as well as of grain weight and grain dry weight per ear to grain and ear numbers for the second dimension, were later confirmed by the correlation network and the correlation matrix shown in Figure 3 and Table 6.

The correlation network showed positive correlations among aboveground biomass, stalk and chaff weights, grain yield, and grain and ear numbers (Figure 3). It must be highlighted the strong correlations of aboveground biomass with grain yield (0.86; Table 6) and stalk weight (0.81), as well as between grain yield and grain number (0.84) and between grain number and ear number (0.75). It is also worth mentioning the negative correlations found between grain weight and the remaining grain yield components, with the highest correlation found between grain weight and grain number (−0.56), while the only positive correlation was found with GYE (0.51). Among the non-mineral nutrient traits, only a remarkable negative correlation between TP and TAC (−0.55), but a positive correlation with TPhC (0.30), must be highlighted. Overall, the B, Cu, Fe, K, Mg, Na, P, and Zn mineral concentrations in grain were positively correlated among them, whereas Ca and S concentrations were negatively correlated with them (excepting K). These mineral concentrations were also negatively correlated with wheat production and grain yield components. Highly negative correlations were found of the aboveground, stalk, and chaff biomasses to the concentrations in grain of Cu, Fe, Mg, and Zn (in a range between −0.28 and −0.60), as well as of Na and P, but with lower correlations (−0.13 to −0.26). Likewise, negative correlations of these mineral concentrations to grain yield components were also observed, with the highest negative correlations specially found between Cu, Fe, Mg, and Zn with grain yield, grain number, ear number, and GYE (−0.14 to −0.49). Nevertheless, Ca, K, and S concentrations showed positive correlations with plant biomass and most of the grain yield components. Both TAC and TPhC were also positively correlated with wheat production and grain yield components, although no consistent correlations with the mineral concentrations were found. The TP was highly negatively correlated with stalk weight (−0.62), GYE (−0.59), S concentration (−0.56), and grain weight (−0.51), and less correlated with the aboveground biomass (−0.39), K concentration (−0.35), chaff weight (−0.26), and yield (−0.15). However, it was positively correlated with grain and ear numbers (0.14 and 0.26, respectively), HI (0.42) and Cu (0.32), Fe (0.25), and Zn (0.46) concentration. There was not a clear tendency for correlations between grain weight and the nutritional quality traits. Besides the negative correlation described above with the TP, the matrix showed the highest negative correlation between grain weight and TPhC (−0.41) concentrations in grain, while the correlations with the mineral concentrations ranged between −0.33 and 0.32.

### 2.5. Grain Nutrient Content

Figures S1 and S2 show the grain nutrient contents expressed as mass of these nutrients per grain (e.g., grain starch content) or in the whole grains per plant (e.g., grain starch yield or grain B uptake). Genotype 41 showed the lowest grain starch and TP content and, together with genotypes 61 and 150, TPhC. Genotypes 95 and 74 had a higher starch content than genotype 41, while genotypes 74 and 23 also showed a higher TP content than genotypes 41, 43, and 61. Genotype 23 exhibited higher TPhC content than genotypes 41, 61, and 150. Likewise, genotypes 23 and 95 also showed higher TAC in the grain than genotype 150. Among genotypes, a similar pattern of changes for grain starch, TP, TPhC, and TAC yields was found, genotypes 41, 43, 61, and 95 showing the greatest values and 8, 74, 76, and 150 the lowest. Genotype 43, as compared to 76, showed the largest starch, TPhC and TAC yields and both genotypes 43 and 61 exhibited higher grain starch yield than 150. TPhC yield was higher in genotype 43 than 76 and, along with genotype 41, higher than genotypes 74 and 150. The TAC yield was higher in genotypes 23, 41, 43, 61, and 95 than 150 and it was also higher in genotypes 41 and 43 than genotype 8.

Regarding the grain mineral contents, a different pattern of changes among genotypes was also found. The grain B, K, and S content remained unchanged. The grain Cu, Fe, Mg, P, and Zn contents were higher for genotypes 8, 23, and 74 than 41, 43, and 61, but only higher than 150 for the grain Mg and P contents. In line with this pattern of changes, the grain Na content was higher for genotype 74 than 43, and the Ca content higher for genotypes 76 and 95 than genotypes 8 and 150. By contrast, greater Ca, Cu, K, Mg, P, S, and Zn uptakes were found for genotypes 41, 43, 61, and 95 than genotypes 8, 74, 76, and 150, exhibiting the same trend observed for the non-mineral nutrient yields. However, the grain B, Fe, and Na uptake did not change among genotypes.

## 3. Discussion

Increases in atmospheric CO_2_ and temperature are likely to modify plant growth and nutrient demand, with the consequent impact on crop productivity and quality.

### 3.1. Grain Yield and Related Traits

The few experiments conducted to investigate the interactive effects of elevated CO_2_ and high temperature reported that the stimulation of crop performance and yield by CO_2_ enrichment was counteracted by increasing temperature [27,30,31]. Therefore, the exploration of genotypic variability might be a promising approach for the selection of improved crop varieties to ensure food security and the improvement of our knowledge on plant production and adaptation to future climatic conditions. Only a limited range of crop germplasm is possible in the rather small size of free air CO_2_ enrichment (FACE) plots [32]. In contrast, a set of 64 wheat varieties grown in the field in ambient CO_2_ was examined relative to growth traits and photosynthetic capacity [33]. Following a screening with 60 wheat lines, here we have compared 10 genotypes for biomass, yield, and nutritional quality under elevated CO_2_ and high temperature, thus providing for significant genetic variation. We found significant differences in the aboveground biomass and grain yield across the 10 wheat genotypes as well as in other yield related traits, although the grain and ear numbers did not differ statistically among genotypes. These results confirm the importance of the evaluation of the genotypic variability on yield performance under a changing climate. Even though grain yield was poorly correlated with grain weight, we observed that grain yield was positively correlated with aboveground biomass and grain and ear numbers and all of them were correlated with each other. These findings suggest that grain yield production was sustained by increased grain number due to a higher number of productive tillers rather than heavier grains. Our data resemble previous work where the increased grain yield by elevated CO_2_ was closely associated with higher grain number per unit ground area due to a higher number of tillers [18,34]. While grain number per ear was also increased in another study, both grain number per unit ground area and grain number per ear contributed to the increase in grain yield due to the fact that ear number was not affected by elevated CO_2_ [35]. In the present work, the most productive genotypes (41, 43, and 61) exhibited higher grain and ear numbers than the less productive ones (8 and 150), as well as a higher grain number per ear as shown by genotype 43. In this regard, it is important to highlight that increases in grain yield due to the implementation of the Green Revolution have been driven mostly by grain number per unit area and ear rather than grain weight [36]. In the case of bread wheat in Spain, genetic improvement of yield from 1930 to 2000 was accounted by an increase in grain number while grain weight remained unchanged [37]. Similarly, grain yield progress was correlated with grain number per square meter, but not with other yield components in the spring wheat breeding program at CIMMYT [38]. The positive relationship between harvest index and grain number and, to a lesser extent, grain yield is in agreement with most studies of yield progress in cereals [38,39], in line with the fact that grain yield is usually related to the grain number per square meter [40], as the most important yield component.

In spite of the yield stimulation induced by elevated CO_2_, higher temperatures accelerate crop phenological development, resulting in a shortened grain filling period and impaired grain yield through a reduction of grain number per ear, ear number and grain weight [41,42,43]. In spring wheat grown under field conditions, Lizana and Calderini [44] applied moderately high temperatures at different growth stages and found varying relationships between reduction in grain yield and grain weight depending on the timing of temperature stress. A negative effect of a post-anthesis heatwave on wheat grain yield associated with decreased grain size was also reported by Weicher et al. [42]. In our study, the negative correlation found between grain number and grain weight could indicate that there is competition between growing grains for limited assimilates. Although that is the most common interpretation, Areche and Slafer [45] proved that grain weight was concomitantly reduced when grain number increased by increasing the proportion of grains that are constitutively smaller in the canopy independently of any competition among grains. Regardless of the origin of the negative relationship, grain size is more heritable and, therefore, less plastic than grain number [46]. In general, genotypes with higher grain yield exhibited a trend towards lower grain weight.

### 3.2. Grain Nutritional Quality Traits

Mineral nutrients play important roles in the biochemical and physiological functions of biological systems. While higher plants obtain their minerals primarily from the soil, animal and humans depend mostly on higher plants to supply them with minerals [47]. Humans require nutrients in adequate amount for proper development and healthy lives. In our study, there were considerable variations in grain protein and mineral nutrient concentrations among wheat genotypes. These results resemble previous findings in two wheat genotypes grown in the field in temperature gradient chambers [20], and they are in good agreement with the well-documented large variation observed in various kinds of wheat and their related species in previous studies under multiple environmental conditions [47,48,49,50]. Variation for both Fe and Zn concentrations did not reach statistical significance, which contrasts with the high variability reported in wild emmer wheat [51], although lower levels of variability for these elements have also been found in old and modern French bread wheats [48].

The amount of minerals in the grain depends on different processes including uptake by the root system, translocation and redistribution within the plant tissues, remobilization to the grain, and accumulation in the developing grain [47]. In the present study, several significant relationships have been identified between grain mineral nutrients, which may indicate the existence of one or more common genetic or physiological mechanisms related to the processes previously mentioned. Thus, we found a strong positive correlation between Fe and Zn, as well as an association of Zn and TP concentration of wheat grains consistent with some previous studies performed on bread wheat [50]. These relationships presumably might be linked to QTLs controlling grain Fe, Zn, and TP concentrations as found in emmer wheat, double haploid populations, and diploid wheat [52,53] and in a recombinant inbred line population derived from a cross between durum wheat and wild emmer [54], although QTL information in bread wheat is limited [55]. Distelfeld et al. [56] suggested that the *Gpc-B1* locus encoding a transcription factor of the NAC family (*NAM-B1*) induces accelerated senescence and contributes to the remobilization of protein, Fe and Zn from leaves to grain, and consequently greater grain concentrations. Uauy et al. [52] discovered that delayed senescence could simultaneously decrease N, Fe, and Zn content in wheat plants, indicating that the remobilization of Fe and Zn is linked to the remobilization of N. Likewise, co-localization of QTLs for Zn and Fe concentrations has been reported in rice [57]. Not only Fe and Zn showed high correlation with each other, but also Cu was highly correlated with them in the current study, in accordance with the results obtained in the work conducted by Pandey et al. [50] on Indian and Turkish bread wheat genotypes. This can be related to a major QTL on chromosome 5 controlling high Fe, Zn, Cu, and Mn content in *Triticum monococcum* genotypes [58]. There was also a relatively high correlation among Mg and micronutrients such as Zn, Fe, and Cu, suggesting physiological coupling of the accumulation processes of minerals in wheat grain. QTLs analysis for cationic mineral concentrations in seeds of *Arabidopsis thaliana* [59] revealed no co-localization of QTLs for Mg, Zn, and Fe. However, in their study the correlations between the three minerals were very low compared to the correlations we observed in bread wheat, as reported previously [48], which may indicate that the accumulation of grain constituents is different in crop species like wheat. Another important relationship was found between P and Mg concentrations, in agreement with other published works with wheat [50,53]. Similarly, positive correlations were found between P and Cu, Fe, K, and, to a lesser extent, with Zn, which were possibly related to the known effect of phytic acid for binding Mg and other cations in grains [48,49,51,55].

Interestingly, we observed a negative correlation between S and grain TP concentration, possibly reflecting a loss of S-containing amino acids. Despite the similarity between nitrate and sulphate assimilatory pathways [60], their regulation in response to the availability of the respective nutrient ions and the environment is different [61]. The observed association is relevant because metabolic proteins (albumin, globulin), which account for 15–20% of the total wheat grain protein, are rich in S-containing amino acids (i.e., cysteine and methionine), as well as in lysin [9,62]. Hence, it is tempting to speculate that a preferential decline of metabolic proteins is likely to make the wheat grain quality poorer with regard to nutritional value, irrespective of any further change in gluten storage proteins responsible for grain processing quality. In our previous work [29], where we investigated the transcriptional response induced by elevated CO_2_ combined with a high temperature in the flag leaf of durum wheat grown in field chambers at ear emergence, the transcript levels for a gene involved in glucosinolate degradation were increased. This result suggests that plants may catabolize glucosinolates to use the released sulfur to assist primary metabolism, such as protein synthesis in the leaf, allowing a readjustment to adverse conditions. Such a finding adds further support to the previous suggestion in the current study with bread wheat grown under similar conditions in growth chambers. Several studies have reported that protein concentration and composition in mature wheat grain are strongly affected by nitrogen and sulfur supply [26,63]. Therefore, further research is needed to assess the grain amino acids and protein composition and the coordination of nitrogen and sulfur metabolism through the development of wheat genotypes under the studied environmental conditions.

Wheat grains are not only a source of proteins and minerals, but also of carbohydrates, vitamins, fibers, and bioactive compounds that are important for human health due to their antioxidant activity [8]. With regard to the starch concentration, as the main C pool in grains, we did not find differences among the bread wheat genotypes studied, whereas variation in TPhC concentration and TAC was observed, as it was reported in a previous study with six wheat genotypes grown at four different locations [64]. Large genotypic variability in the TPhC has also been observed in earlier reports in wheat [65,66,67], although variation related to environmental conditions seems to be larger than genotypic differences [66]. In comparison between high yielding and low yielding genotypes, genotypes 41 and 43 had the highest concentration of TPhC, whereas genotype 150 had the lowest, suggesting that it may be possible to select genotypes enriched in bioactive compounds with benefits to the health of consumers.

### 3.3. Grain Yield and Quality Trade-Off

Although much work has been done to assess the effects of elevated CO_2_ or temperature on wheat regarding yield and quality, comparatively little attention has been paid to the relevance of the plant biomass, grain yield, and grain nutritional quality traits relationships when both factors are applied simultaneously to explore the genotypic variability.

In the current experiment, the maximum variability explained by the genotypic variation was highly associated with the *Wheat production* components (i.e., aboveground, stalk, and chaff biomasses) and the *Mineral nutrients* in the grain (B, Ca, Cu, Fe, K, Mg, Na, P, S, and Zn; Figure 2a, Table 5), providing evidence of plant biomass relevance for the nutritional quality of the grain. Nevertheless, the first dimension of both variable groups showed opposite correlations for the first dimension of the MFA (Figure 2b), suggesting a trade-off between plant biomass and mineral composition in the grain. Among the *Yield components* and the *Non-mineral nutrients* traits, a lesser contribution, but still with a similar opposite relationship, was found with TP and grain yield as the most related traits. Thus, the genotypes with higher biomass production (41, 43, 61, and 95) showed the highest grain yield, grain and ear numbers and grain Ca, K and S concentrations, but the lowest concentrations in the grain for TP, Cu, Fe, Mg, and Zn. These findings suggest that increased wheat biomass and yield can be counteracted by the altered chemical composition of the grain, leading to reduced quality [9]. In line with this, several studies have reported a decline of macro and microelements under elevated CO_2_ [9,17,19,20], with differences depending on genotypes, exposure system, and rooting volume. Likewise, the opposite relationship between grain yield and grain TP concentration resembles previous findings in wheat grown under elevated CO_2_ since CO_2_ yield stimulation has been linked to decreased grain protein concentration [9,12,16,17]. Explanations for the decline in protein concentration include N dilution by increased concentrations of non-structural carbohydrates, restricted N uptake due to decreased transpiration, and N assimilation inhibition or even other unclear mechanisms [12,14,15,16,29]. Although little information about the effects of CO_2_ on macro and microelements in wheat grains is known [9], a dilution of grain components as a consequence of CO_2_-stimulated carbohydrate production has also been proposed [17]. In agreement with our results, other studies have often found negative associations between grain yield and grain protein concentration, indicating that the dilution of N compounds in grain of genotypes was a consequence of the breeding process [68]. Similarly, evidence for a negative relationship between grain yield and grain mineral nutrient concentrations is well documented, pointing to modern wheat cultivars with greater yield capacity having lower grain mineral concentrations than the old varieties with lower yield [8,49,69].

In our study, the most productive genotypes (41, 43, 61, and 95) exhibited an increasing trend in grain Cu, Mg, P, and Zn uptakes that were accompanied by lower concentrations of those minerals, while the least productive ones showed the opposite trend. Although these results might be consistent with a possible dilution effect due to higher biomass, the high yielding genotypes also showed a higher grain starch yield, while the starch concentration remained unchanged. Therefore, these findings seem inconsistent with the mineral dilution by an accumulation of carbohydrates operating alone, which cannot explain this trade-off between minerals and biomass to any large extent. Interestingly, Myers et al. [17], using a meta-analysis approach, suggested that dilution cannot be the only reason for the decrease in grain mineral concentrations under CO_2_ enrichment because the extent of the decline in concentration varies between different nutrients. In line with this proposal, we have observed a similar trend to a more marked increase of grain Ca, Cu, K, Mg, P, and S uptakes in the most productive genotypes, which was accompanied by higher concentrations of Ca, K, and S but lower concentrations of Cu, P, or Mg. The general negative correlations of the Cu, Fe, Mg, Na, P, and Zn concentrations with the aboveground, stalk, and chaff biomasses, grain yield, and grain and ear numbers suggest that other mechanisms more complex than dilution could also be involved, such as nutrient uptake, distribution or translocation to the grain.

Similarly, the decrease in grain TP concentration in the high yielding genotypes (41, 43, and 61) was accompanied by lower grain TP content but higher grain TP yield. These results suggest that although these genotypes were able to take up more N and they had higher grain TP yield [70,71], the increase in biomass accumulation could be larger than the increase in N acquisition [72]. Thus, the decrease in grain protein concentration can be partially attributed to dilution effect due to increased grain yield [12,73]. In this sense, it is worth nothing that apart from the associations described above, the MFA (Table 5) showed that for the second dimension the TP concentration was positively correlated with HI and grain and ear numbers, and all of them negatively correlated with grain weight. Our results indicate that the TP concentration is mainly and negatively associated with improved plant biomass and grain yield, whereas an amelioration in the decline of grain TP concentration might be associated with greater grain yield based on higher grain and ear numbers rather than heavier grains. Therefore, the selection of wheat varieties with greater grain and ear numbers could be used as a strategy for the improvement of grain yield and offset any loss of grain TP concentration, contributing to the maintenance of the wheat grain nutritional quality in the future climatic scenario. Hence, the dilution hypothesis cannot be fully supported since the yield of TP and minerals are enhanced in the highest yielding genotypes under combined elevated CO_2_ and temperature, but possibly to a lesser extent than grain yield. Other features could be considered, such as their ability to store and distribute minerals in the vegetative tissues or to scavenge them from the soil prior to redistribution to the grain. All these processes may be likely altered by elevated CO_2_ and high temperature applied simultaneously, making it difficult to draw any further conclusions.

Finally, our study provides information on the nutritional profile of the genotypes and shows that the two least productive genotypes (8 and 150) exhibited higher grain TP concentration than the three highest productive ones (41, 43, and 61). This suggests that improved grain protein nutritional quality can be achieved at the cost of lower yield, which is accompanied by lower grain mineral nutrient concentrations and total antioxidant capacity, particularly in genotype 150 (Gazul). Several genotypes contained high concentrations of certain minerals as well as phenolic compounds. Thus, genotype 41 can be selected as that combining superior grain yield with comparably high nutritional quality characteristics because it is a high yielding genotype with slightly lower total grain protein concentration, which is compensated by the enrichment of most of the mineral nutrients and bioactive compounds as well as a higher total antioxidant capacity, both with beneficial effects on human health.

## 4. Materials and Methods

### 4.1. Plant Material and Growth Conditions

**Figure A1:**
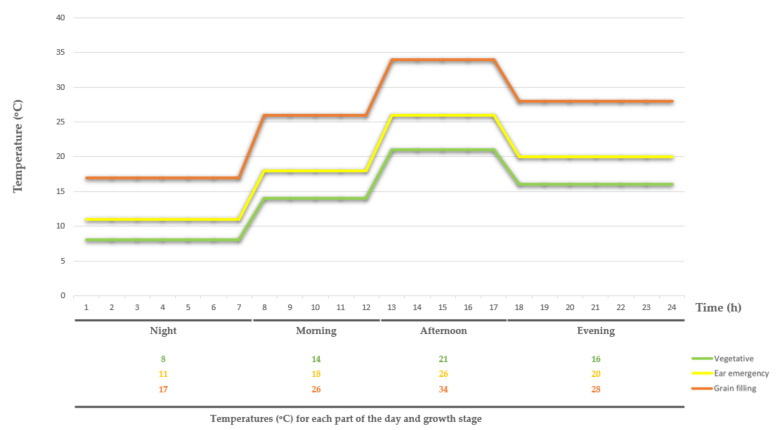
Schematic representation of the daily and seasonal oscillations of temperatures simulated throughout wheat development.

The experiment was conducted with 10 bread wheat genotypes (*Triticum aestivum*), using nine genotypes (referred to as lines 8, 23, 41, 43, 61, 74, 76, 94) of the *Heat Tolerance Wheat Screening Nursery* (8HT HTWSN) collection of the CIMMYT [74], together with the Gazul genotype (referred to as line 150) with high yield and adaptability to the Mediterranean climate of Salamanca region (Spain) [13,75] (see Table A1). The genotypes of the 8HT HTWSN collection were selected following a survey of 60 lines of this collection for growth and yield under the same environment as in the present study. Seeds were sown in 5L pots with 1.2 kg of peat:perlite (4:1) substrate, with a density of five plants per pot after emergence. Four grams of KNO_3_ and 4 g of KH_2_PO_4_ were added to each pot, with the peat providing a sufficient provision of other nutrients [76]. Pots were placed in controlled environment chambers (3.6 m length × 4.8 m width × 2.4 m height) maintained on a 16/8 light/dark hour regime with an irradiance of 400 µmol m^−2^ s^−1^ at the top of the canopy, provided by a combination of blue-plus red-peak fluorescent lamps, and relative humidity of 40%/60% day/night. The atmospheric CO_2_ concentration was set at 700 µmol mol^−1^ by injecting pure CO_2_ [77,78]. The temperature was 4 °C above-current temperatures simulating the daily and seasonal oscillations of typical temperatures in natural environments of the Salamanca region. Four different sections were established to reproduce the daily temperature oscillations: night and initial, central, and final parts of the photoperiod. These temperatures were increased by three levels reproducing the natural seasonal oscillations throughout wheat development (see Figure A1). The experiment was a completely randomized design with five replicates (pot) per each of the studied genotypes. Water was supplied during crop development three times per week to maintain pot field capacity, and the pots were rotated twice a week to avoid edge effects.

### 4.2. Harvesting and Yield Parameter Measurements

At maturity, the aboveground plant parts were harvested from each pot and divided into stalks (stems and leaves) and ears. Grains and chaff components were separated from the ears by manual threshing. The number of ears and grains per plant and per ear were determined, and the dry weights for the stalk, chaff, and the grain yield per plant and per ear were recorded after drying in an oven at 60 °C for 48 h. The grain weight was estimated as the quotient between the grain yield and the grain number per plant. The harvest index (HI) was calculated as the ratio of grain yield to total aboveground biomass.

### 4.3. Sample Preparation and Analysis of Starch

Wheat grains were ground into whole meal flour using a mill (IKA Micro Fine Mill Grinder Culatti MFC, Germany). To quantify the grain starch content, an aliquot of 30 mg of the ground grain material was successively extracted with 80% ethanol HEPES–KOH (pH 7.5) at 80 °C and water at 60 °C, then pooling the extracts. Starch was determined in the insoluble residue from the extraction after incubation with amyloglucosidase and α-amylase at 37 °C overnight. Then, starch was measured spectrophotometrically with an assay coupled to NADP^+^ reduction reaction as described by Morcuende et al. [79].

### 4.4. Total N and Protein Concentration

After Kjeldahl digestion of dried and ground grain material with H_2_SO_4_ using a Se catalyst, the pH was adjusted to 3–4 using 1 M triethanolamine buffer (pH 7.2) and 5 M KOH as required. Nitrogen was determined (Ammonia Rapid kit, Megazyme, Ireland) through the glutamate dehydrogenase catalyzed conversion of NH_4_^+^ and 2-oxoglutarate to L-glutamate, the NADP^+^ reduction being recorded spectrophotometrically at 340 nm. The total protein concentration was calculated by multiplying the concentration of N by a conversion factor of 5.7 for wheat grain [18].

### 4.5. Total Antioxidant Capacity and Total Phenolic Compound Measurements

The measurements of both total antioxidant capacity and total phenolic compounds were made in a multimodal 96-well plate reader (FLUOstart Omega, BMG Labtech, Ortenberg, Germany) using the ferric ion reducing antioxidant power (FRAP) and the Folin-Ciocolteau colorimetric methods, respectively [80,81].

### 4.6. Determination of Mineral Nutrients

For the grain mineral nutrients assay, about 100 mg of the dried and ground grain material were mixed with 5 mL of 65% HNO_3_ and 2 mL of 35% hydrogen peroxide in a Teflon container and heated in a MWS-3+ microwave digestion system (Berghof Products + Instruments GmbH). Afterwards, the digested solution was diluted to 20 mL by adding deionized water [50]. The concentration for the macro and microelements (S, P, B, K, Ca, Cu, Fe, Mg, Na, and Zn) was determined in an ICP-OES Optima 7000 DV with a radial configuration. The grain content of each mineral was also calculated.

### 4.7. Statistical Analysis

The experiment was conducted in a completely randomized design with five replicates per genotype. A one-way analysis of variance (ANOVA) was performed using the package *stats* from the statistical software *R* [82]. Previously, the normality and homoscedasticity of the data were examined using the Levene test. For each trait studied, comparisons of all possible pairs of means among genotypes were conducted through the Tukey’s honest significant difference post-hoc test. When unequal variances were detected, the Welch adjustment for ANOVA (Welch test) [83] was applied and all-pairs comparisons were determined using Tamhane’s T2 test [84]. The canonical biplot and the multiple factor analysis were carried out using *MultBiplotR*, *FactoMineR* and *factoextra* [85,86,87]. To assess the effects of the variation in the vegetative plant biomass and the grain yield components over the nutritional quality traits, the variables studied in the present experiment were split up in four groups (*Wheat production*, *Yield components*, *Non-mineral nutrients,* and *Mineral nutrients*). The Correlation Network was performed with *psych* [88] along with the software *Cytoscape* [89], using a threshold for the Spearman’s correlation values of *r* ≥ |0.45|. For the whole study, differences were considered statistically significant at *p* < 0.05.

## 5. Conclusions

The expected global rise in the atmospheric CO_2_ concentration in association with higher mean temperatures and other extreme climatic events is threatening the resilience of current food systems. Therefore, improved crop varieties that can withstand these challenges will be required to ensure food security in the face of an endless growing worldwide population. To our knowledge, this is the first attempt to explore the performance of wheat genotypic variability under combined elevated CO_2_ and high temperature for the improvement of grain yield and grain nutritional quality and their relationships. Several 8HT HTWSN lines outyielded the local variety Gazul, showing that adaptation to the future environment can be enhanced through plant breeding. The increased grain yield was related to an increase in grain and ear numbers rather than an increase in grain weight. The results give valuable insights into the physiological processes modulating wheat responses in the future climate scenario. Most of the associations among different nutrients were in accordance with previously conducted nutritional analyses under different environmental conditions. With this regard, the novel findings in this study indicate that further research will be helpful to understand the coordination of nitrogen and sulfur metabolism and their implications in grain mineral nutrient concentrations under the studied environmental conditions. Grain protein concentration was negatively correlated with plant biomass and yield-related traits, while mineral nutrients appear to be mainly affected by plant biomass. Variation in the nutritional profile among genotypes can be useful for selecting genotypes with promising nutritional concentrations.

## Figures and Tables

**Table 1:** Production (aboveground, stalk and chaff biomasses) and grain yield components (grain yield, grain number, ear number, grain weight, grain yield per ear, grain number per ear, and harvest index) of 10 wheat genotypes grown under elevated CO_2_ and high temperature.

**Genotype**	**Aboveground** (g plant^−1^)				**Stalk** (g plant^−1^)				**Chaff** (g plant^−1^)				**Grain Yield** (g plant^−1^)				**Grain Number** (No. plant^−1^)			
**8**	19.78	±	1.77	**b**	8.39	±	0.52	**ab**	3.26	±	0.33	**ab**	8.13	±	1.27	**ac**	207.89	±	35	**a**
**23**	20.61	±	2.52	**ab**	8.18	±	0.70	**ab**	3.14	±	0.68	**ab**	9.30	±	1.25	**abc**	229.82	±	35	**a**
**41**	23.12	±	1.24	**ab**	8.93	±	0.56	**ab**	3.92	±	0.35	**ab**	10.27	±	0.95	**ab**	308.75	±	64	**a**
**43**	24.41	±	2.75	**ab**	9.40	±	1.59	**ab**	4.18	±	0.39	**a**	10.83	±	1.08	**b**	281.74	±	22.1	**a**
**61**	24.90	±	2.08	**a**	10.44	±	1.16	**a**	4.47	±	0.65	**ab**	9.98	±	0.73	**abc**	287.37	±	41.8	**a**
**74**	20.78	±	2.97	**ab**	9.30	±	1.81	**ab**	2.77	±	0.48	**b**	8.71	±	1.54	**abc**	202.84	±	39.2	**a**
**76**	21.42	±	2.85	**ab**	9.64	±	1.19	**ab**	3.51	±	0.69	**ab**	8.27	±	1.26	**ac**	205.46	±	37.5	**a**
**94**	20.53	±	2.11	**ab**	8.45	±	1.49	**ab**	3.53	±	0.38	**ab**	8.56	±	0.59	**ac**	240.53	±	29.9	**a**
**95**	22.25	±	2.25	**ab**	9.18	±	1.05	**ab**	3.60	±	0.39	**ab**	9.48	±	1.02	**abc**	230.08	±	23.1	**a**
**150**	19.46	±	2.52	**b**	7.71	±	1.32	**b**	3.79	±	0.80	**ab**	7.96	±	0.59	**c**	220.05	±	22.4	**a**
**Mean**	21.73	±	2.80		8.96	±	1.34		3.62	±	0.68		9.15	±	1.35		241.45	±	49.30	
***p* value**	0.005				0.044				0.008 *				0.001				0.007 *			
	**Ear number** (No. plant^−1^)				**Grain weight** (mg grain^−1^)				**GYE** (g ear^−1^)				**GNE** (No. ear^−1^)				**HI**			
**8**	6.15	±	0.65	**a**	39.33	±	3.79	**ab**	1.32	±	0.11	**b**	33.63	±	2.4	**c**	0.41	±	0.03	**ab**
**23**	5.90	±	1.07	**a**	40.58	±	1.58	**ab**	1.59	±	0.12	**abc**	39.14	±	1.47	**abc**	0.45	±	0.01	**a**
**41**	7.60	±	1.27	**a**	34.03	±	4.73	**ab**	1.37	±	0.13	**ab**	40.43	±	2.86	**ab**	0.44	±	0.02	**ab**
**43**	6.35	±	0.42	**a**	38.58	±	4.35	**ab**	1.71	±	0.16	**c**	44.59	±	5.31	**a**	0.44	±	0.03	**ab**
**61**	7.05	±	0.74	**a**	35.16	±	4.11	**ab**	1.42	±	0.1	**abc**	40.70	±	3.38	**a**	0.40	±	0.03	**ab**
**74**	6.00	±	0.85	**a**	43.09	±	1.63	**a**	1.45	±	0.14	**abc**	33.78	±	3.83	**bc**	0.42	±	0.05	**ab**
**76**	6.20	±	1.46	**a**	40.49	±	2.7	**ab**	1.37	±	0.21	**ab**	33.65	±	3.44	**c**	0.39	±	0.02	**b**
**94**	5.90	±	0.76	**a**	35.88	±	3.53	**ab**	1.47	±	0.18	**abc**	40.92	±	3.7	**a**	0.42	±	0.04	**ab**
**95**	5.85	±	0.52	**a**	41.21	±	1.96	**ab**	1.62	±	0.05	**ac**	39.30	±	0.87	**abc**	0.43	±	0.01	**ab**
**150**	5.65	±	0.72	**a**	36.30	±	2.38	**b**	1.42	±	0.15	**abc**	39.07	±	1.84	**abc**	0.41	±	0.03	**ab**
**Mean**	6.27	±	1.00		38.46	±	4.11		1.47	±	0.18		38.52	±	4.55		0.42	±	0.03	
***p* value**	0.176 *				0.004 *				0.001				7.37 × 10^−6^				0.001 *			
*GNE*: grain number ear^−1^; *GYE*: grain yield ear^−1^; HI: harvest index. Each value is the mean ± standard deviation (SD) of five replicates (*n* = 5) for each genotype. *Mean* indicates the mean ± SD for each trait with all the genotypes and replicates (N = 50). The calculation of statistical significance (*p* value) is based on one-way analysis of variance (ANOVA) or Welch test (*). Within columns, numbers followed by the same letter indicate non-statistically significant differences at *p <* 0.05 as determined by post-hoc tests.																	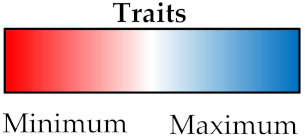			

**Table 2:** Non-mineral nutrients (starch and total protein concentrations, total antioxidant capacity and total phenolic compound concentration) in wheat grains of 10 wheat genotypes grown under elevated CO_2_ and high temperature.

**Genotype**	**Starch** (µmol g^−1^)				**TP** (mg g^−1^)				**TAC** (µmol eq Trolox g^−1^)				**TPhC** (µmol eq Galic Ac. g^−1^)			
**8**	3589.29	±	213.82	**a**	94.93	±	13.43	**ab**	1.19	±	0.21	**ab**	6.23	±	0.61	**ab**
**23**	3272.55	±	197.88	**a**	86.23	±	2.66	**a**	1.35	±	0.12	**a**	6.51	±	0.41	**a**
**41**	3356.48	±	123.52	**a**	80.09	±	11.35	**ab**	1.40	±	0.20	**a**	6.25	±	0.62	**a**
**43**	3661.72	±	330.58	**a**	77.72	±	8.43	**ab**	1.35	±	0.15	**a**	6.26	±	0.25	**a**
**61**	3515.85	±	170.01	**a**	83.11	±	12.12	**ab**	1.25	±	0.18	**ab**	6.03	±	0.49	**ab**
**74**	3507.61	±	89.15	**a**	83.00	±	6.00	**ab**	1.26	±	0.17	**ab**	5.27	±	0.33	**b**
**76**	3310.92	±	108.57	**a**	81.47	±	7.35	**ab**	1.30	±	0.11	**a**	6.03	±	0.55	**ab**
**94**	3448.82	±	263.92	**a**	85.86	±	17.54	**ab**	1.39	±	0.18	**a**	6.10	±	0.40	**ab**
**95**	3690.14	±	330.48	**a**	74.79	±	3.79	**b**	1.43	±	0.09	**a**	6.29	±	0.53	**a**
**150**	3446.49	±	233.64	**a**	90.63	±	7.29	**ab**	0.97	±	0.10	**b**	5.82	±	0.15	**ab**
**Mean**	3479.99	±	241.88		83.78	±	10.68		1.29	±	0.19		6.08	±	0.53	
***p* value**	0.060				0.013 *				0.002				0.013			
*TAC*: total antioxidant capacity; *TP*: total protein; *TPhC*: total phenolic compounds. Each value is the mean ± standard deviation (SD) of five replicates (*n* = 5) for each genotype. *Mean* indicates the mean ± SD for each trait with all the genotypes and replicates (N = 50). The calculation of statistical significance (*p* value) is based on one-way analysis of variance (ANOVA) or Welch test (*). Within columns, numbers followed by the same letter indicate non-statistically significant differences at *p <* 0.05 as determined by post-hoc tests.												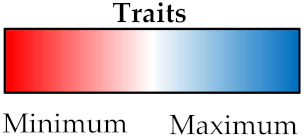				

**Table 3:** Mineral nutrient (B, Ca, Cu, Fe, K, Mg, Na, P, S and Zn) concentrations of 10 wheat genotypes grown under elevated CO_2_ and high temperature.

**Genotype**	**B** (µg g^−1^)				**Ca** (µg g^−1^)				**Cu** (µg g^−1^)				Fe (µg g^−1^)				**K** (µg g^−1^)			
**8**	2.14	±	0.53	**a**	280.37	±	23.24	**c**	6.92	±	0.42	**a**	22.76	±	2.95	**a**	3436.69	±	203.07	**a**
**23**	1.67	±	0.42	**a**	317.57	±	33.71	**abc**	6.43	±	0.83	**abc**	21.77	±	2.12	**a**	3410.14	±	98.87	**a**
**41**	1.56	±	0.62	**a**	388.19	±	32.76	**d**	6.56	±	0.55	**ab**	21.21	±	1.69	**a**	4097.08	±	152.49	**b**
**43**	1.19	±	0.33	**a**	330.20	±	8.44	**abcd**	5.66	±	0.38	**bc**	17.98	±	2.28	**a**	3644.07	±	240.32	**ac**
**61**	1.98	±	0.34	**a**	347.96	±	18.93	**abd**	6.36	±	0.90	**abc**	19.57	±	2.21	**a**	3921.67	±	188.12	**bc**
**74**	1.53	±	0.65	**a**	309.98	±	37.66	**abc**	6.50	±	0.61	**abc**	24.18	±	8.14	**a**	3487.24	±	190.11	**a**
**76**	1.27	±	0.24	**a**	339.24	±	48.76	**abcd**	5.30	±	0.47	**c**	19.34	±	1.11	**a**	3681.37	±	167.47	**ac**
**94**	1.33	±	0.35	**a**	357.77	±	31.20	**abd**	6.35	±	0.57	**abc**	21.36	±	2.17	**a**	3393.57	±	148.70	**a**
**95**	1.33	±	0.24	**a**	364.81	±	14.56	**bd**	5.90	±	0.49	**abc**	19.37	±	1.99	**a**	3716.26	±	131.68	**ac**
**150**	1.70	±	1.01	**a**	302.97	±	17.26	**ac**	6.21	±	0.20	**abc**	20.92	±	1.97	**a**	3484.80	±	163.20	**a**
**Mean**	1.57	±	0.56		333.91	±	40.58		6.22	±	0.69		20.85	±	3.43		3627.29	±	273.80	
***p* value**	0.049 *				2.11 × 10^−5^				0.004				0.152				2.01 × 10^−7^			
	**Mg** (µg g^−1^)				**Na** (µg g^−1^)				**P** (µg g^−1^)				**S** (µg g^−1^)				**Zn** (µg g^−1^)			
**8**	1314.49	±	48.84	**a**	14.37	±	5.99	**a**	5488.15	±	212.79	**ab**	33.97	±	31.37	**a**	35.47	±	3.04	**a**
**23**	1322.06	±	82.92	**a**	12.42	±	5.82	**a**	5359.80	±	160.00	**ab**	41.83	±	15.25	**a**	37.67	±	2.28	**a**
**41**	1276.31	±	63.32	**ab**	14.38	±	9.53	**a**	5603.18	±	143.59	**a**	101.52	±	33.43	**b**	35.00	±	3.44	**a**
**43**	1194.65	±	69.17	**ab**	3.74	±	1.56	**a**	5309.50	±	235.44	**ab**	76.01	±	26.39	**ab**	31.38	±	3.55	**a**
**61**	1152.71	±	17.78	**b**	13.22	±	9.19	**a**	5447.82	±	97.35	**ab**	77.37	±	19.35	**ab**	34.06	±	3.73	**a**
**74**	1303.19	±	79.88	**ab**	7.44	±	1.09	**a**	5388.58	±	353.24	**ab**	74.24	±	21.05	**ab**	38.67	±	5.72	**a**
**76**	1176.51	±	96.62	**ab**	18.06	±	12.50	**a**	5312.23	±	141.24	**ab**	76.60	±	13.21	**ab**	32.68	±	2.70	**a**
**94**	1295.18	±	69.67	**ab**	12.44	±	5.27	**a**	5284.17	±	256.79	**ab**	66.56	±	9.92	**ab**	37.79	±	5.32	**a**
**95**	1232.46	±	69.16	**ab**	9.94	±	6.24	**a**	5424.47	±	195.71	**ab**	90.37	±	15.18	**b**	33.51	±	2.27	**a**
**150**	1172.26	±	110.51	**ab**	6.38	±	1.90	**a**	5045.06	±	305.33	**b**	64.56	±	15.59	**ab**	37.31	±	2.76	**a**
**Mean**	1243.98	±	92.35		11.24	±	7.48		5366.30	±	246.71		70.30	±	27.42		35.35	±	4.06	
***p* value**	0.001				0.006 *				0.042				0.001				0.038			
Each value is the mean ± standard deviation (SD) of five replicates (*n* = 5) for each genotype. *Mean* indicates the mean ± SD for each trait with all the genotypes and replicates (N = 50). The calculation of statistical significance (*p* value) is based on one-way analysis of variance (ANOVA) or Welch test (*). Within columns, numbers followed by the same letter indicate non-statistically significant differences at *p <* 0.05 as determined by post-hoc tests.																	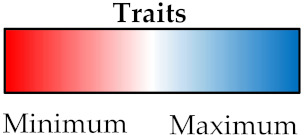			

**Table 4:** Correlations between original variables (the wheat production, grain yield, non-mineral, and mineral nutrient traits) and the canonical axis.

Dim.1			Dim.2		
Traits	Corr.	Cos2	Traits	Corr.	Cos2
K	0.72	0.52	Chaff	0.41	0.17
Ca	0.62	0.39	B	0.35	0.12
Grain number	0.59	0.35	GNE	0.27	0.07
S	0.59	0.35	Grain number	0.27	0.07
Grain yield	0.59	0.35	Cu	0.25	0.06
Aboveground	0.58	0.34	TP	0.25	0.06
Chaff	0.49	0.24	Starch	0.18	0.03
GNE	0.44	0.20	TPhC	0.14	0.02
Ear number	0.41	0.17	K	0.13	0.02
TAC	0.40	0.16	Ear number	0.12	0.02
Stalk	0.36	0.13	Grain yield	0.08	0.01
P	0.28	0.08	Aboveground	0.08	0.01
GYE	0.21	0.04	HI	0.03	0.00
TPhC	0.21	0.04	P	−0.07	0.00
HI	0.15	0.02	Zn	−0.08	0.01
Starch	0.14	0.02	Fe	−0.09	0.01
Na	0.01	0.00	GYE	−0.09	0.01
B	−0.23	0.05	Stalk	−0.13	0.02
Grain weight	−0.25	0.06	Na	−0.15	0.02
Cu	−0.28	0.08	S	−0.17	0.03
Mg	−0.29	0.08	Mg	−0.22	0.05
Fe	−0.36	0.13	Ca	−0.23	0.05
Zn	−0.44	0.20	Grain weight	−0.41	0.17
TP	−0.45	0.20	TAC	−0.42	0.17
*Corr.*: correlation; *Dim.*: Dimension; *GNE*: grain number ear^−1^; *GYE*: grain yield ear^−1^; *HI*: harvest index; *TAC*: total antioxidant capacity; *TP*: total protein; *TPhC*: total phenolic compounds. *Corr.* indicates the correlation between the variable and the dimension. The squared correlation (*Cos2*) values between the variables and the dimensions are used to estimate the quality of the representation.					
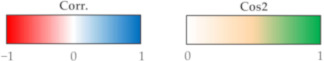					

**Table 5:** Correlations and contributions between the original variables, the variable groups and the supplementary group with the first two dimensions of the multiple factorial analysis.

Dim. 1				Dim. 2			
Variable Groups	Corr.	Cos2	Contr.	Variable Groups	Corr.	Cos2	Contr.
Wheat production	0.81	0.63	36.17	Wheat production	0.08	0.01	4.79
Yield components	0.37	0.10	16.35	Yield components	0.74	0.40	46.01
Non-mineral nutrients	0.39	0.08	17.49	Non-mineral nutrients	0.60	0.20	37.41
Mineral nutrients	0.67	0.22	29.99	Mineral nutrients	0.19	0.02	11.80
**Supplementary group**				**Supplementary group**			
Genotype	0.55	0.03		Genotype	0.27	0.01	
**Continuous variables**				**Continuous variables**			
Aboveground	0.91	0.83	15.26	Grain number	0.79	0.62	10.91
Chaff	0.77	0.59	10.83	TPhC	0.72	0.52	20.05
Grain yield	0.77	0.59	7.43	Ear number	0.68	0.46	8.01
Stalk	0.74	0.55	10.07	TP	0.64	0.41	15.55
Grain number	0.53	0.28	3.49	HI	0.60	0.36	6.26
S	0.53	0.28	4.15	Ca	0.54	0.29	5.97
Ear number	0.45	0.20	2.59	GNE	0.42	0.18	3.15
TAC	0.45	0.20	5.57	Grain yield	0.42	0.18	3.11
K	0.38	0.15	2.20	Chaff	0.28	0.08	2.04
GYE	0.35	0.12	1.54	P	0.24	0.06	1.19
Ca	0.32	0.10	1.53	Cu	0.21	0.04	0.89
GNE	0.31	0.10	1.21	Zn	0.15	0.02	0.49
Starch	0.16	0.02	0.67	K	0.14	0.02	0.40
Grain weight	0.04	0.00	0.02	Aboveground	0.13	0.02	0.42
TPhC	0.02	0.00	0.01	Na	0.11	0.01	0.25
Na	−0.02	0.00	0.01	Fe	−0.05	0.00	0.06
B	−0.03	0.00	0.01	Starch	−0.13	0.02	0.65
HI	−0.08	0.01	0.08	B	−0.15	0.02	0.45
P	−0.26	0.07	1.00	TAC	−0.17	0.03	1.17
Mg	−0.48	0.23	3.41	Mg	−0.22	0.05	0.97
Cu	−0.52	0.27	4.01	S	−0.24	0.06	1.16
Fe	−0.57	0.33	4.92	Stalk	−0.30	0.09	2.33
TP	−0.64	0.41	11.25	GYE	−0.35	0.13	2.20
Zn	−0.76	0.58	8.75	Grain weight	−0.84	0.71	12.37
*Dim.*: dimension; *Contr.*: contribution; *Corr*.: correlation*; GNE*: grain number ear^−1^; *GYE*: grain yield ear^−1^; *HI*: harvest index; *TAC*: total antioxidant capacity; *TP*: total protein; *TPhC*: total phenolic compounds. *Genotype* is the group based on a categorical variable specifying the genotypic identity of each sample. The vegetative biomass, grain yield and nutritional quality traits were split up into four groups: *Wheat production* (aboveground, stalk and chaff biomasses), *Yield components* (grain yield, grain number, ear number, grain weight, grain yield ear^−1^, grain number ear^−1^, and harvest index), *Non-mineral nutrients* (starch, total protein, total phenolic compound concentrations and total antioxidant capacity) and *Mineral nutrients* (B, Ca, Cu, Fe, K, Mg, Na, P, S, and Zn mineral concentrations). *Corr.* indicates the correlation between the variable and the dimension. The values for the squared correlation (*Cos2*) between the variables and the dimensions are used to estimate the quality of the representation. *Contr.* expresses the contributions, in percentage, of each variable in accounting for the variability in the dimension.							
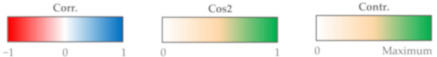							

**Table 6:** Correlation coefficient matrix for the wheat production, grain yield, non-mineral, and mineral nutrient components among of 10 wheat genotypes grown under elevated CO_2_ and high temperature.

	Stalk	Chaff	Grain Yield	Grain Number	Ear Number	Grain Weight	GYE	GNE	HI	Starch	TP	TAC	TPhC	B	Ca	Cu	Fe	K	Mg	Na	P	S	Zn
Aboveground	**0.81**	**0.75**	**0.86**	**0.66**	**0.63**	0.02	0.20	0.22	−0.03	0.09	**−0.39**	0.24	0.07	0.05	0.29	**−0.35**	**−0.47**	0.26	**−0.45**	−0.19	−0.20	**0.37**	**−0.60**
Stalk		**0.50**	**0.46**	0.19	0.33	0.34	0.19	−0.08	**−0.50**	0.13	**−0.62**	0.29	−0.21	0.13	0.06	−0.31	−0.32	0.32	−0.28	−0.13	−0.13	**0.44**	**−0.54**
Chaff			**0.61**	**0.64**	**0.55**	−0.34	0.01	0.30	−0.13	−0.09	−0.26	0.05	0.16	0.04	0.24	−0.30	**−0.50**	0.28	**−0.59**	−0.14	−0.26	**0.38**	**−0.50**
Grain yield				**0.84**	**0.69**	−0.11	0.24	**0.37**	**0.44**	0.08	−0.15	0.27	0.26	−0.06	**0.42**	−0.30	**−0.47**	0.11	−0.34	−0.16	−0.14	0.19	**−0.49**
Grain number					**0.75**	**−0.56**	−0.05	**0.55**	**0.50**	0.01	0.14	0.05	**0.40**	−0.09	**0.48**	−0.18	**−0.38**	0.14	**−0.42**	−0.16	−0.07	0.07	−0.31
Ear number						**−0.41**	**−0.48**	−0.05	0.22	−0.13	0.26	−0.01	0.28	0.03	0.31	−0.14	**−0.36**	0.16	−0.35	0.05	0.05	0.13	−0.29
Grain weight							**0.51**	**−0.42**	−0.28	0.13	**−0.51**	0.24	**−0.41**	0.05	−0.33	−0.13	0.06	−0.15	0.32	0.04	−0.09	0.17	−0.11
GYE								**0.46**	0.13	0.22	**−0.59**	**0.37**	−0.06	−0.04	−0.02	−0.27	−0.16	−0.12	0.01	−0.26	−0.31	0.10	−0.25
GNE									**0.43**	0.09	−0.08	0.13	0.30	−0.08	0.33	−0.05	−0.08	0.14	−0.20	−0.32	−0.10	0.00	0.00
HI										−0.01	**0.42**	0.10	**0.35**	−0.27	0.31	0.07	−0.03	−0.21	0.13	−0.11	0.09	−0.27	0.14
Starch											−0.10	0.05	−0.05	−0.05	−0.25	−0.03	−0.19	−0.07	−0.06	−0.12	−0.17	−0.13	−0.32
TP												**−0.55**	0.30	−0.03	−0.10	0.32	0.25	**−0.35**	0.01	0.12	0.11	**−0.56**	**0.46**
TAC													0.02	−0.04	0.28	−0.16	−0.28	0.17	0.21	0.07	0.01	**0.35**	**−0.35**
TPhC														−0.08	0.27	0.15	0.02	0.04	−0.12	0.07	0.15	−0.26	0.01
B															−0.25	0.31	0.21	0.18	−0.03	0.27	0.18	0.19	0.09
Ca																−0.16	−0.14	**0.37**	−0.08	−0.01	0.24	0.26	−0.07
Cu																	**0.76**	0.08	**0.47**	0.24	**0.48**	−0.20	**0.65**
Fe																		0.11	**0.50**	0.25	**0.53**	−0.19	**0.78**
K																			−0.16	0.05	**0.59**	**0.57**	−0.05
Mg																				0.10	**0.46**	−0.18	**0.45**
Na																					0.13	0.02	0.13
P																						0.14	**0.44**
S																							−0.24
*GNE*: grain number ear^−1^; *GYE*: grain yield ear^−1^; *HI*: harvest index; *TAC*: total antioxidant capacity; *TP*: total protein; *TPhC*: total phenolic compounds. Data were generated from Spearman correlation analysis. Values in bold represent signification (*p <* 0.05).																				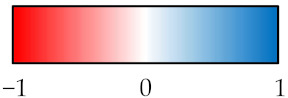			

## Data Availability

Data are available upon request from the corresponding authors.

## Supplementary Materials

The following are available online at https://www.mdpi.com/article/10.3390/plants10061043/s1, Figure S1: Boxplots of non-mineral nutrients per grain and plant, Figure S2: Boxplots of the contents in mineral components per grain and plant, Table S1: Eigenvalues and explained variance for the canonical biplot.

## Appendix A

**Table A1:** Catalogue of the 10 wheat genotypes employed in the present study. Pedigree, accession code and top name of genotypes belonging to the *Heat Tolerance Wheat Screening Nursery* (8HT HTWSN) collection of the International Maize and Wheat Improvement Centre (CIMMYT) are provided.

Genotype	Pedigree	Accesion	Top Name
8	KACHU/KIRITATI	BW 49924	CMSS07Y00127S-0B-099Y-099M-099NJ-099NJ-6WGY-0B
23	SUPER 152*2/TECUE #1	BW 49956	CMSS07B00614T-099TOPY-099M-099Y-099M-49WGY-0B
41	SUPER 152/BAJ #1	BW 50048	CMSS07Y00195S-0B-099Y-099M-099Y-5M-0WGY
43	SUPER 152//WEEBILL1*2/BRAMBLING	BW 50050	CMSS07Y00196S-0B-099Y-099M-099Y-6M-0WGY
61	TOBARITO M 97/PASTOR*2//AKURI	BW 50122	CMSS07Y01094T-099TOPM-099Y-099M-099NJ-099NJ-17WGY-0B
74	WEEBILL1/KUKUNA//TACUPETO F2001/3/QUAIU #2	BW 50193	CMSS07B00246S-099M-099Y-099M-5WGY-0B
76	WHEATEAR/KUKUNA/3/C80.1/3*BATAVIA//2*WEEBILL1/4/QUAIU	BW 50196	CMSS07B00264S-099M-099NJ-099NJ-2WGY-0B
94	WEEBILL1*2/KURUKU*2//SUPER 152	BW 50264	CMSS07B00685T-099TOPY-099M-099Y-099M-17WGY-0B
95	FRET2/KUKUNA//FRET2/3/HEILO/4/BLOUK #1	BW 50266	CMSS07B00715T-099TOPY-099M-099Y-099M-7WGY-0B
150	Gazul		
