# Supplementary material for: Genotypic Variability on Grain Yield and Grain Nutritional Quality Characteristics of Wheat Grown under Elevated CO2 and High Temperature
Source: Plants (Basel). 2021 May 21;10(6):1043. doi: 10.3390/plants10061043 (PMC8224326; doi:10.3390/plants10061043)
Supplement: Supplementary file 1 [file plants-10-01043-s001.zip › plants-1204485-supplementary.pdf]

Table S1. Eigenvalues and explained variance for the canonical biplot.

Dimension	Eigenvalue	Explained variance	Cumulative
1	3.98	32.45	32.45
2	3.18	20.73	53.18
3	2.80	16.07	69.26
4	2.36	11.39	80.65
5	1.74	6.22	86.87
6	1.67	5.75	92.62
7	1.38	3.89	96.51
8	1.09	2.45	98.96
9	0.71	1.04	100.00

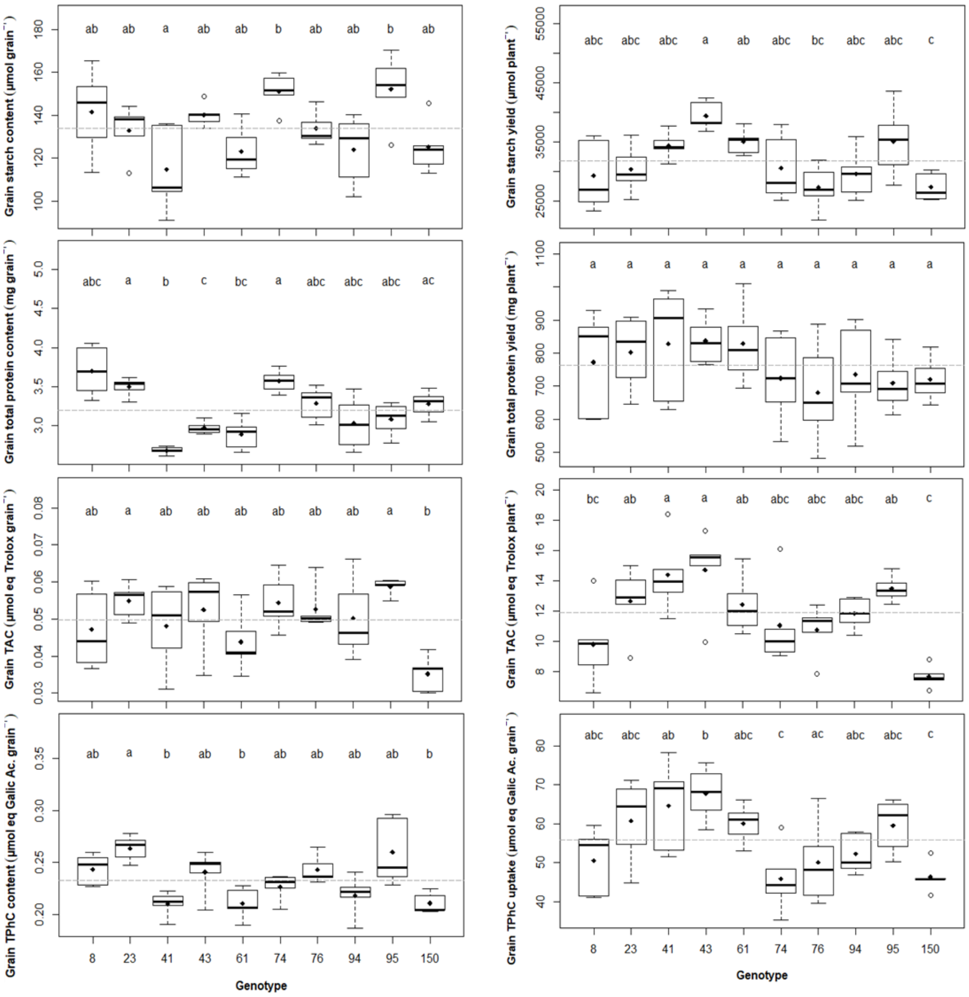

Figure S1. Boxplots of non-mineral nutrients per grain and plant.

TAC: total antioxidant capacity; *TP*: total protein; *TPhC*: Total phenolic compounds.

Black dots represent the mean of the five replicates (*n*=5) per genotype. The grey dotted line represents the mean among all the genotypes and replicates (*N*=50). The same letter indicates non-statistically significant differences at $p < 0.05$ as determined by post-hoc tests.

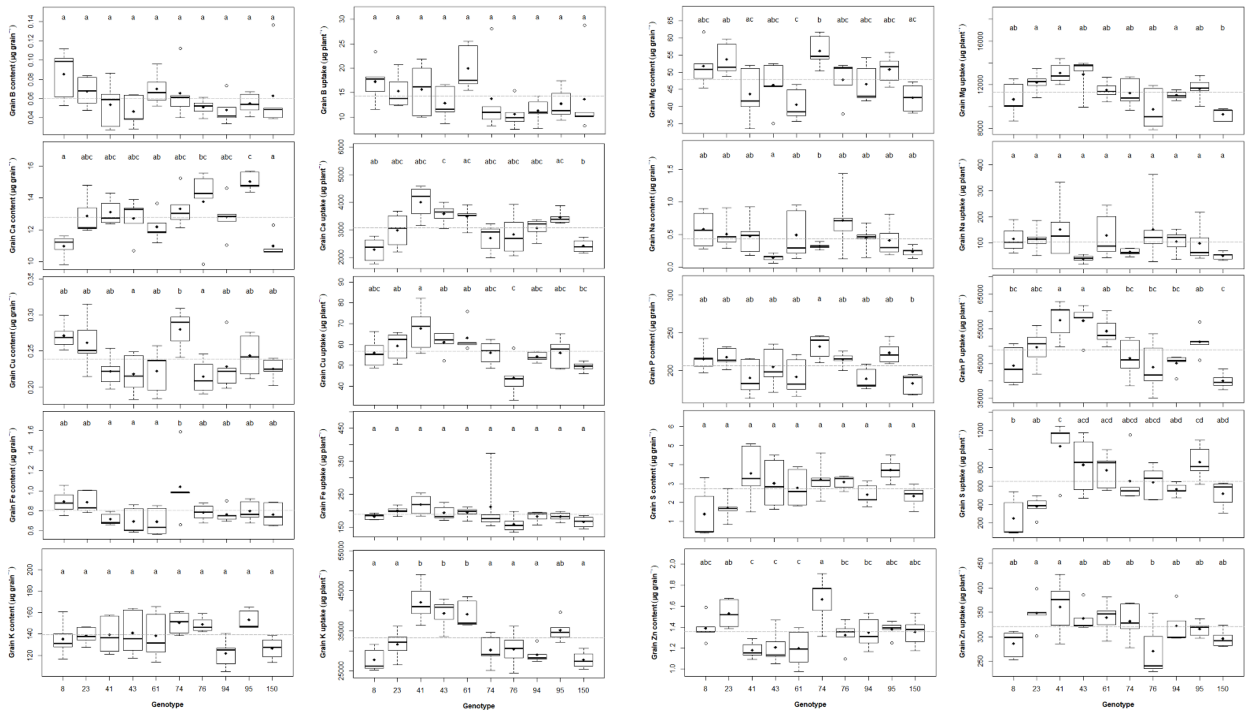

Figure S2. Boxplots of the contents in mineral components per grain and plant.

Black dots represent the mean of the five replicates (n=5) per genotype. The grey dotted line represents the mean among all the genotypes and replicates (N=50). The same letter indicates non-statistically significant differences at $p < 0.05$ as determined by post-hoc tests.
